# Correlates of blood pressure and blood glucose screenings in Cameroon: insights from the 2018 Demographic and Health Survey

**DOI:** 10.1093/inthealth/ihab033

**Published:** 2021-06-12

**Authors:** Precious Adade Duodu, Pascal Agbadi, Henry Ofori Duah, Ernest Darkwah, Jerry John Nutor

**Affiliations:** Department of Nursing and Midwifery, School of Human and Health Sciences, University of Huddersfield, Queensgate, Huddersfield, HD1 3DH, UK; Department of Nursing, College of Health Sciences, Kwame Nkrumah University of Science and Technology, Kumasi, Ghana; Research Department, FOCOS Orthopaedic Hospital, Accra, Ghana; Department of Psychology, University of Ghana, P. O. Box LG 84, Legon, Ghana; Department of Family Health Care Nursing, School of Nursing, University of California, San Francisco, CA, USA

**Keywords:** Cameroon, diabetes mellitus, hypertension, screening

## Abstract

**Background:**

Hypertension and diabetes, two major risk factors for cardiometabolic diseases, are associated with high morbidity and mortality rates. Early detection through screening can initiate early treatment to reduce adverse outcomes. The current study sought to investigate the correlates of blood pressure and blood glucose screenings in Cameroon.

**Methods:**

We used secondary data from the 2018 Cameroon Demographic and Health Survey. Adjusting for a complex sample design, we performed multivariate prevalence ratio estimates of the blood pressure and blood glucose screenings.

**Results:**

Approximately 60% and 30% of Cameroonians had undergone blood pressure and blood glucose screenings, respectively. More females (68%) had undergone blood pressure screenings compared with their male counterparts (44.1%). In the multivariate model, gender, age, education, marital status, household wealth index and region of residence were significantly associated with both blood pressure and blood glucose screenings in the full sample. Previous blood pressure screening was associated with an increased likelihood of blood glucose screening and vice versa. A modification effect of gender was observed in the association between the correlates and both outcomes.

**Conclusion:**

Our findings uncovered individuals with a decreased likelihood for blood pressure and blood glucose screenings and this can inform policy decisions to ensure targeted screening aimed at early detection and management.

## Introduction

Hypertension and diabetes mellitus are major risk factors for cardiovascular and renal diseases, and are associated with significant morbidity and mortality.^1,2^ Rapid sociodemographic changes have spurred shifts in the risk factors for hypertension and diabetes, including inactivity and sedentary lifestyles, poor dietary habits, stress, family history and gender.^1,3,4^ In 2020, about 1.13 billion people globally were living with hypertension. Similarly, 422 million adults were living with diabetes and this is projected to reach 642 million by 2035, with the majority living in low- and middle-income countries (LMICs).^5^ In 2017, cardiovascular diseases and diabetes accounted for 17.4% of the global disease burden.^3,6^ Non-communicable diseases (NCDs) are the second most common cause of deaths in sub-Saharan Africa (SSA), accounting for 2.6 million deaths.^7^ Therefore, there is a need for increased attention to the dangers of hypertension, diabetes and other NCDs in SSA.^8^ Available data suggest that the majority of people with hypertension and diabetes in SSA are unaware of their status, and are rarely treated, putting them at high risk for stroke, heart and renal diseases.^9,10^

The factors that affect hypertension and diabetes management are multifaceted, including policy-, health system-, healthcare professional- and patient-related factors.^9^ Research and the treatment of most NCDs are poorly funded by donors and governments, with only 2% of the cumulative health development assistance allocated to NCDs in 2017.^6,11^ For instance, the Cameroon government's health expenditure was 0.6% of the gross domestic product in 2017.^12^ Over 74% of countries in SSA including Cameroon have no guidelines for the management of hypertension.^9^ Cameroon, a Central African country, is home to over 25 million people.^13^ In 2019, the prevalence of age-adjusted (20–79 y) diabetes was 6.0%, which is higher than that of Africa (3.9%) but lower than the global prevalence of 9.3%.^5^ The prevalence of hypertension spans from 5.7% (rural) and 21.9% (semiurban) to 47.5% (urban milieu), with a national average of 31.0%.^14^ Age, male gender, diabetes and obesity are reported predictors of hypertension.^8,9^ Reportedly, hypertension and diabetes prevalence, awareness, treatment and control rates across population subgroups in Cameroon are unknown.^15^

To improve access to affordable healthcare for those with these conditions, a collaborative effort is required to improve early detection.^15^ Community-based interventions such as screenings for hypertension and diabetes are simple, sustainable, effective and cost-effective prevention and control measures associated with improved outcomes.^6,16^ The inadequate and ineffective screening and timely management regimens have resulted in the high prevalence of these diseases in LMICs. There is a paucity of literature on the predictors of blood pressure and blood glucose screenings in Cameroon. Therefore, the current study estimated the prevalence and predictors of blood pressure and blood glucose screenings in Cameroon among people aged ≥15 y using the 2018 Cameroon Demographic Health Survey (CDHS) datasets.

## Methods

### Design and study sample

We used datasets from the cross-sectional CDHS conducted in 2018. A two-stage sampling design structured the 2018 CDHS. The response rates were 98.2% and 97.5% for males and females, respectively.^17^ A comprehensive methodological report on the CDHS has been published elsewhere.^17^ We merged the female and the male datasets. The female dataset (weighted) contained 14 677 women within the age group of 15–64 y. One thousand and sixty-one cases in the female dataset had no records on the outcome variables; therefore, they were removed from the female dataset. The female study sample underlying the results of the present study consisted of 13 616 weighted cases. The male dataset (weighted) contained 6978 men within the age group of 15–60 y. The total weighted study sample comprised 20 594 cases. These cases were nested within 430 primary sampling units (PSU), with 237 in urban and 193 in rural areas. The 430 sampling units were further nested within 21 strata.

### Measures

#### Outcome

The two outcome variables for the study were blood pressure and blood glucose screenings. In the datasets, blood glucose screening was measured by asking each respondent the question: ‘Ever had blood glucose measured by a doctor or nurse?’ Blood pressure screening was measured by asking each respondent the question: ‘Ever had blood pressure measured?’ The responses to these questions were either ‘Yes’ or ‘No.’

#### Explanatory variables

Based on literature review^18,19^ and availability of variables in the datasets, the following explanatory variables were selected: gender, age, education, marital status, household wealth, rural-urban residence and region of residence. Except for the age variable, the remaining variables were used as measured and categorised in the datasets. The age variable was recoded as 15–24, 25–34, 35–44 and ≥45 y. The CDHS computed the household wealth index using principal component analysis (PCA) to assign weights to each household's assets and calculated cumulative scores from the assigned weights. From the PCA results, the CDHS categorised the households into five wealth quintiles: poorest, poorer, middle, rich and richest. The following were the household's assets used in the calculation: household characteristics (source of drinking water, type of toilet, sharing of toilet facilities, the main material for the roof, walls and floors, as well as type of cooking fuel, among other household characteristics) and household possessions and assets (ownership of a television, radio, vehicle, bicycles, motorcycles, watches, agricultural land, farm animals/livestock and bank account, among others).

#### Data analysis

All data analyses were performed in STATA version 14 (Release 14, College Station, TX: StataCorp LP). First, weighted frequencies and percentages were used to estimate summary statistics for the sample characteristics. We assessed the interaction effect of gender on the relationship between each study explanatory variable and blood pressure and blood glucose screenings among Cameroonians. We assessed the interaction effect by performing a Wald χ^2^ test, pegging statistical significance at p≤0.05.

Based on the results of the interaction effect assessment, we built separate adjusted and unadjusted models for the female sample, the male sample and the full sample. The analyses were performed after adjusting for the sample design (PSU and sample strata) and weights. In STATA, the ‘svy’ command was used to set the analytical environment in line with DHS recommendation. Although the outcomes were dichotomous, we used Poisson regression to estimate prevalence ratios. The rationale and the choice of Poisson regression to estimate prevalence ratios for dichotomous outcomes for cross-sectional data are sufficiently explained elsewhere.^20–22^ The default ‘svy’ computes the standard errors (SEs) by using the linearised variance estimator called first-order Taylor linearisation. This procedure eliminated the incorrect estimation of the SEs associated with the confidence intervals (CI) of the regression coefficients. Before using Poisson regression, we assessed the assumption of the overdispersion of the data using the ‘nbreg’ command. The assessment indicated that the likelihood ratio (LR) test of alpha=0 and the p-value of the LR test was >0.05. This suggests that the conditional variance is equal to the conditional mean, making Poisson an appropriate model fit for the study outcomes.

## Results

### Study sample characteristics

About 6 out of 10 people (60%) in Cameroon have been screened for blood pressure and 3 out of 10 (30%) have been tested for blood glucose levels by a nurse or doctor. More females than males have been tested for blood pressure (males=44.1%; females=68.0%) and blood glucose (males=20.1%; females=34.5%). The majority of respondents were aged 15–24 y (40.7%), had attained secondary education (47.3%), were currently married (53.6%) and lived in urban areas (55.3%). Details of the sample characteristics are reported in Table 1_Supplementary Data.

### Gender modifies the association between each correlate and blood pressure screening

All the study variables were significantly associated with blood glucose screening in a bivariate analysis (unadjusted; Table 1); therefore, they were all included in a multivariable model (adjusted; Table 2). Further, interaction effect analyses were performed to ascertain whether gender modifies the relationship between each correlate and blood pressure screening (Table 1). The results revealed that gender modifies the relationship between blood pressure screening and the following study variables: blood glucose screening (p<0.001), age (p<0.001), education (p<0.001), marital status (p<0.001), household wealth index (p<0.001), rural-urban residence status (p<0.001) and region of residence (p<0.001) (Table 1). The interaction effect statistics are reported in the last column of Table 1.

**Table 1.** Correlates of blood pressure screening among Cameroonians by gender (bivariate)

	Females		Males		Interaction effect
Correlates	PR [95% CI]	%^A^	PR [95% CI]	%^A^	p-value
Blood glucose ever tested					F(3,407)=509.50; p<0.001
No	1	52.38	1	31.73	
Yes	1.86^***^ [1.78 to 1.95]	97.62	2.95^***^ [2.75 to 3.16]	93.51	
Age, y					F(7,403)=161.89; p<0.001
15–24	1	50.15	1	29.31	
25–34	1.62^***^ [1.56 to 1.69]	81.45	1.64^***^ [1.48 to 1.81]	47.97	
35–44	1.62^***^ [1.55 to 1.69]	81.20	1.78^***^ [1.63 to 1.94]	52.11	
≥45	1.55^***^ [1.46 to 1.65]	77.67	2.11^***^ [1.93 to 2.31]	61.77	
Education					F(7,403)=80.89; p<0.001
None	1	59.33	1		
Primary	1.21^***^ [1.13 to 1.30]	71.78	1.83^***^ [1.53 to 2.20]	21.92	
Secondary	1.13^**^ [1.04 to 1.21]	66.80	2.07^***^ [1.72 to 2.49]	40.12	
Higher	1.43^***^ [1.32 to 1.55]	84.80	3.26^***^ [2.70 to 3.95]	45.43	
Marital status					F(5,405)=175.80; p<0.001
Never married	1	46.53	1	34.67	
Currently married	1.70^***^ [1.63 to 1.78]	79.16	1.53^***^ [1.42 to 1.64]	53.04	
Previously married	1.72^***^ [1.63 to 1.81]	80.00	1.50^***^ [1.31 to 1.71]	51.91	
Household wealth					F(9,401)=48.39; p<0.001
Poorest	1	55.06	1	17.43	
Poorer	1.19^***^ [1.09 to 1.31]	65.74	2.07^***^ [1.65 to 2.59]	36.07	
Middle	1.26^***^ [1.14 to 1.39]	69.22	2.53^***^ [2.04 to 3.13]	44.06	
Richer	1.32^***^ [1.19 to 1.47]	72.68	3.00^***^ [2.42 to 3.72]	52.26	
Richest	1.33^***^ [1.20 to 1.48]	73.37	3.46^***^ [2.77 to 4.31]	60.22	
Rural-urban residence					F(3,407)=106.21; p<0.001
Rural	1	64.20	1	34.37	
Urban	1.11^***^ [1.05 to 1.16]	71.05	1.51^***^ [1.36 to 1.69]	52.06	
Region of residence					F(23,387)=31.75; p<0.001
Adamawa	1	53.37	1	29.24	
Centre (without Yaounde)	1.39^***^ [1.18 to 1.63]	74.04	1.98^***^ [1.57 to 2.50]	57.86	
Douala	1.40^***^ [1.19 to 1.65]	74.62	1.73^***^ [1.33 to 2.26]	50.68	
East	1.38^***^ [1.16 to 1.65]	73.68	1.43^**^ [1.09 to 1.86]	41.75	
Far-North	0.99 [0.82 to 1.18]	52.64	0.84 [0.62 to 1.13]	24.55	
Littoral (without Douala)	1.35^***^ [1.14 to 1.60]	72.08	1.58^***^ [1.23 to 2.04]	46.27	
North	1.28^***^ [1.05 to 1.54]	68.06	0.68^**^ [0.51 to 0.90]	19.74	
North-West	1.28^*^ [1.07 to 1.53]	68.15	1.12 [0.84 to 1.50]	32.84	
West	1.43^**^ [1.22 to 1.69]	76.52	2.05^***^ [1.63 to 2.57]	59.93	
South	1.11 [0.94 to 1.31]	59.40	1.64^***^ [1.28 to 2.09]	47.91	
South-West	1.17 [0.97 to 1.41]	62.58	0.84 [0.55 to 1.29]	24.52	
Yaounde	1.39^***^ [1.17 to 1.64]	74.01	2.45^***^ [1.95 to 3.08]	71.71	

PR, prevalence ratio.

^A^ estimated blood pressure screening prevalence of the population segment: this was used to estimate the prevalence ratios.

Exponentiated coefficients; 95% CIs in brackets.

^*^p≤0.05, ^**^p≤0.01, ^***^p≤0.001.

**Table 2.** Associated factors of blood pressure screening in a gender-stratified multivariate model

	Females	Males	Full sample
Correlates	APR [95% CI]	APR [95% CI]	APR [95% CI]
Gender			
Male			1
Female			1.38^***^ [ 1.32 to 1.45]
Blood glucose ever tested			
No	1	1	1
Yes	1.63^***^ [1.57 to 1.69]	2.16^***^ [2.00 to 2.32]	1.76^***^ [1.69 to 1.82]
Age, y			
15–24	1	1	1
25–34	1.22^***^ [1.18 to 1.26]	1.41^***^ [1.30 to 1.54]	1.23^***^ [1.18 to 1.27]
35–44	1.17^***^ [1.13 to 1.21]	1.48^***^ [1.34 to 1.63]	1.18^***^ [1.14 to 1.22]
≥45	1.11^***^ [1.05 to 1.17]	1.61^***^ [1.45 to 1.79]	1.20^***^ [1.15 to 1.82]
Education			
None	1	1	1
Primary	1.09^**^ [1.03 to 1.15]	1.30^**^ [1.09 to 1.55]	1.04 [0.98 to 1.11]
Secondary	1.10^**^ [1.04 to 1.17]	1.40^***^ [1.17 to 1.67]	1.06 [1.00 to 1.13]
Higher	1.30^***^ [1.21 to 1.39]	1.63^***^ [1.35 to 1.96]	1.28^***^ [1.19 to 1.38]
Marital status			
Never married	1	1	1
Currently married	1.49^***^ [1.44 to 1.56]	1.09^*^ [1.00 to 1.19]	1.43^***^ [1.38 to 1.48]
Previously married	1.51^***^ [1.43 to 1.58]	1.05 [0.93 to 1.17]	1.42^***^ [1.36 to 1.49]
Household wealth			
Poorest	1	1	1
Poorer	1.12^**^ [1.03 to 1.22]	1.43^**^ [1.14 to 1.80]	1.17^***^ [1.07 to 1.27]
Middle	1.16^**^ [1.07 to 1.27]	1.52^***^ [1.21 to 1.91]	1.23^***^ [1.12 to 1.34]
Richer	1.19^***^ [1.09 to 1.30]	1.57^***^ [1.25 to 1.97]	1.26^***^ [1.15 to 1.38]
Richest	1.15^*^ [1.04 to 1.26]	1.61^***^ [1.27 to 2.04]	1.23^***^ [1.12 to 1.36]
Rural-urban residence			
Rural	1	1	1
Urban	1.11 [0.93 to 1.02]	1.06 [0.97 to 1.16]	0.99 [0.94 to 1.03]
Region of residence			
Adamawa	1	1	1
Centre (without Yaounde)	1.25^***^ [1.09 to 1.35]	1.65^***^ [1.35 to 2.00]	1.37^***^ [1.20 to 1.57]
Douala	1.10 [0.95 to 1.27]	1.07 [0.87 to 1.31]	1.10 [0.96 to 1.27]
East	1.22^*^ [1.04 to 1.42]	1.38^**^ [1.12 to 1.69]	1.24^**^ [1.08 to 1.43]
Far-North	1.01 [0.85 to 1.19]	1.02 [0.79 to 1.32]	1.00 [0.86 to 1.17]
Littoral (without Douala)	1.19^*^ [1.03 to 1.37]	1.18 [0.97 to 1.45]	1.21^**^ [1.05 to 1.39]
North	1.37^***^ [1.15 to 1.62]	0.84 [0.66 to 1.07]	1.26^**^ [1.07 to 1.48]
North-West	1.20^*^ [1.04 to 1.40]	1.09 [0.86 to 1.38]	1.20^**^ [1.04 to 1.38]
West	1.19^*^ [1.03 to 1.37]	1.45^***^ [1.22 to 1.72]	1.27^**^ [1.11 to 1.45]
South	1.06 [0.91 to 1.22]	1.44^***^ [1.18 to 1.77]	1.15^*^ [1.01 to 1.32]
South-West	1.05 [0.90 to 1.23]	0.63^**^ [0.45 to 0.88]	0.96 [0.82 to 1.11]
Yaounde	1.17^*^ [1.02 to 1.36]	1.52^***^ [1.27 to 1.82]	1.29^***^ [1.12 to 1.48]

APR, adjusted prevalence ratio.

Exponentiated coefficients; 95% CIs in brackets

^*^p≤0.05, ^**^p≤0.01, ^***^p≤0.001.

### Associated factors of blood pressure screening in a gender-stratified and full-sample multivariate model

In the full-sample multivariate model, gender, blood glucose screening, age, education, marital status, household wealth index and region of residence were significantly associated with blood pressure screening (Table 2). In the gender-stratified sample models, differences existed in the effect of the associated factors on blood pressure screening. For instance, the effect of blood glucose screening on blood pressure screening, although the relationship goes in the same direction, was slightly larger for males (adjusted prevalence ratio [APR]=2.16, 95% CI 2.00 to 2.32) than females (APR=1.63, 95% CI 1.57 to 1.69) (Table 2). The effect of the following associated factors was also slightly larger for males than females, except for marital status: age, education, household wealth index and region of residence (Table 2). For instance, compared with respondents within the 15–24 y age group, respondents within the age groups of 25–34 y (men: APR=1.41, 95% CI 1.30 to 1.54 vs women: APR=1.22, 95% CI 1.18 to 1.26), 35–44 y (men: APR=1.48, 95% CI 1.34 to 1.63 vs women: APR=1.17, 95% CI 1.13 to 1.21) or ≥45 y (men: APR=1.61, 95% CI 1.45 to 1.79 vs women: APR=1.11, 95% CI 1.05 to 1.17) had a higher likelihood of undergoing blood pressure screening (Table 2). For both men and women, the likelihood of blood pressure screening increased with level of education and household wealth. Gender differences were observed in regional variations in blood pressure screening. Rural-urban residence status was not significantly associated with blood pressure screening in either the full or the gender-stratified samples (Table 2).

### Gender modifies the association between each correlate and blood glucose screening

All the study variables were significantly associated with blood glucose screening in the bivariate analysis (unadjusted; Table 3); therefore, they were all included in the multivariable model (adjusted; Table 4). Further, interaction effect analyses were performed to ascertain whether gender modifies the relationship between each correlate and blood glucose screening (Table 3). The results revealed that gender modified the relationship between blood glucose screening and the following study variables: blood pressure screening (p<0.001), age (p<0.001), education (p<0.001), marital status (p<0.001), household wealth index (p<0.001), rural-urban residence status (p<0.001) and region of residence (p<0.001). The interaction effect statistics are reported in the last column of Table 3.

**Table 3.** Correlates of blood glucose screening among Cameroonians by gender (bivariate)

	Females		Males		Interaction effect
Correlates	PR [95% CI]	%^A^	PR [95% CI]	%^A^	p-value
Blood pressure ever screened					F(3,407)=323.23; p<0.001
No	1	2.56	1	2.33	
Yes	19.32^***^ [14.32 to 26.06]	49.53	18.23^***^ [14.05 to 23.66]	42.57	
Age, y					F(7,403)=104.28; p<0.001
15–24	1	20.54	1	10.60	
25–34	2.12^***^ [1.94 to 2.31]	43.56	1.89^***^ [1.55 to 2.31]	20.04	
35–44	2.23^***^ [2.07 to 2.44]	45.84	2.38^***^ [1.96 to 2.89]	25.20	
≥45	2.25^***^ [2.03 to 2.51]	46.32	3.29^***^ [2.73 to 3.96]	34.84	
Education					F(7,403)=74.70; p<0.001
None	1	17.44	1	8.07	
Primary	1.90^***^ [1.62 to 2.23]	33.16	1.99^***^ [1.37 to 2.87]	16.03	
Secondary	2.24^***^ [1.93 to 2.60]	39.00	2.55^***^ [1.77 to 3.68]	20.59	
Higher	3.32^***^ [2.83 to 3.89]	57.83	5.02^***^ [3.49 to 7.22]	40.47	
Marital status					F(5,405)=68.17; p<0.001
Never married	1	23.94	1	13.68	
Currently married	1.68^***^ [1.54 to 1.83]	40.23	1.94^***^ [1.69 to 2.22]	26.50	
Previously married	1.62^***^ [1.44 to 1.82]	38.83	1.58^***^ [1.22 to 2.06]	21.65	
Household wealth					F(9,401)=60.80; p<0.001
Poorest	1	14.97	1	4.51	
Poorer	1.78^***^ [1.47 to 2.17]	26.67	2.24^***^ [1.45 to 3.46]	10.09	
Middle	1.09^***^ [1.72 to 2.54]	31.37	3.89^***^ [2.59 to 5.85]	17.54	
Richer	2.77^***^ [2.30 to 3.35]	41.58	5.71^***^ [3.82 to 8.53]	25.74	
Richest	3.35^***^ [2.79 to 4.03]	50.20	7.73^***^ [5.20 to 11.50]	34.84	
Rural-urban					F(3,407)=116.18; p<0.001
Rural	1	23.85	1	11.18	
Urban	1.81^***^ [1.65 to 1.98]	43.08	2.44^***^ [2.04 to 2.93]	27.32	
Region					F(23,387)=33.76; p<0.001
Adamawa	1	18.21	1	11.45	
Centre (without Yaounde)	1.79^***^ [1.25 to 2.55]	32.60	1.72^**^ [1.15 to 2.55]	19.66	
Douala	3.20^***^ [2.28 to 4.47]	58.19	3.13^***^ [2.10 to 4.67]	35.86	
East	2.38^***^ [1.65 to 3.43]	21.38	0.90 [0.56 to 1.45]	10.30	
Far-North	1.17 [0.82 to 1.67]	39.54	0.71 [0.46 to 1.11]	8.18	
Littoral (without Douala)	2.17^***^ [1.52 to 3.10]	39.54	2.08^***^ [1.43 to 3.04]	23.87	
North	0.67 [0.43 to 1.04]	12.20	0.48^***^[0.30 to 0.77]	5.49	
North-West	1.45 [0.99 to 2.11]	26.35	0.76 [0.45 to 1.30]	8.75	
West	2.69^***^ [1.90 to 3.80]	48.92	3.18^***^ [2.17 to 4.65]	36.39	
South	1.64^**^ [1.15 to 2.33]	29.83	0.84 [0.50 to 1.42]	9.62	
South-West	2.25^***^ [1.55 to 3.26]	40.95	1.58 [0.89 to 2.80]	18.11	
Yaounde	2.46^***^ [1.74 to 3.46]	44.73	3.16^***^ [2.10 to 4.76]	36.20	

PR, prevalence ratio

^A^ Estimated blood pressure screening prevalence of the population segment: this was used to estimate the prevalence ratios.

Exponentiated coefficients; 95% CIs in brackets

^*^p≤0.05, ^**^p≤0.01, ^***^p≤0.001.

### Associated factors of blood glucose screening in a gender-stratified and full-sample multivariate model

In the full-sample multivariate model, gender, blood pressure screening, age, education, marital status, household wealth index, urban-rural residence and region of residence were significantly associated with blood glucose screening (Table 4). In the gender-stratified sample models, differences existed in the effect of the associated factors on blood glucose screening (Table 4). For instance, the effect of blood pressure screening on blood glucose screening, although the relationship goes in the same direction, was slightly larger for females (APR=15.03, 95% CI 11.20 to 20.17) than males (APR=12.50, 95% CI 9.49 to 16.47) (Table 4). However, the effect of age, marital status or household wealth on blood glucose screening was slightly larger for males than females (Table 4). For example, compared with respondents who have never married, respondents who were currently married (men: APR=1.18, 95% CI 1.02 to 1.36 vs women: APR=1.12, 95% CI 1.05 to 1.19) had a higher probability of screening for blood glucose. Rural-urban residence status (with urban residents having a higher likelihood of screening for blood glucose) was significantly associated with blood pressure screening in the female sample, but not in that composed of males (Table 4).

**Table 4.** Associated factors of blood glucose screening in a gender-stratified multivariate model

	Females	Males	Full sample
Correlates	APR [95% CI]	APR [95% CI]	APR [95% CI]
Gender			
Male			1
Female			1.34^***^ [1.24 to 1.44]
Blood pressure ever screened			
No	1	1	1
Yes	15.03^***^ [11.20 to 20.17]	12.50^***^ [9.49 to 16.47]	14.09^***^ [11.54 to 17.19]
Age, y			
15–24	1	1	1
25–34	1.35^***^ [1.25 to 1.26]	1.18^*^ [1.00 to 1.40]	1.31^***^ [1.22 to 1.39]
35–44	1.44^***^ [1.34 to 1.55]	1.39^***^ [1.14 to 1.68]	1.25^***^ [1.10 to 1.43]
≥45	1.50^***^ [1.37 to 1.65]	1.65^***^ [1.34 to 2.02]	1.37^***^ [1.20 to 1.56]
Education			
None	1	1	1
Primary	1.13 [0.99 to 1.30]	1.00 [0.76 to 1.30]	1.11 [0.98 to 1.26]
Secondary	1.31^***^ [1.14 to 1.50]	1.08 [0.82 to 1.41]	1.25^***^ [1.10 to 1.43]
Higher	1.39^***^ [1.20 to 1.61]	1.21 [0.92 to 1.60]	1.37^***^ [1.20 to 1.56]
Marital status			
Never married	1	1	1
Currently married	1.12^***^ [1.05 to 1.19]	1.18^*^ [1.02 to 1.36]	1.13^***^ [1.07 to 1.21]
Previously married	1.02 [0.92 to 1.13]	1.04 [0.75 to 1.31]	1.03 [0.93 to 1.12]
Household wealth			
Poorest	1	1	1
Poorer	1.13 [0.94 to 1.36]	1.11 [0.75 to 1.63]	1.11 [0.93 to 1.33]
Middle	1.14 [0.94 to 1.38]	1.47^*^ [1.01 to 2.14]	1.18 [0.98 to 1.42]
Richer	1.23^*^ [1.01 to 1.49]	1.64^*^ [1.12 to 2.42]	1.29^**^ [3.82 to 1.55]
Richest	1.30^**^ [1.07 to 1.59]	1.79^**^ [1.20 to 2.68]	1.38^***^ [1.15 to 1.66]
Rural-urban residence			
Rural	1	1	1
Urban	1.14^*^ [1.02 to 1.27]	1.13 [0.95 to 1.34]	1.14^**^ [1.03 to 1.25]
Region of residence			
Adamawa	1	1	1
Centre (without Yaounde)	1.25 [0.94 to 1.66]	1.05 [0.77 to 1.43]	1.20 [0.93 to 1.55]
Douala	1.74^***^ [1.33 to 2.26]	1.57^**^ [1.16 to 2.13]	1.71^***^ [1.34 to 2.17]
East	1.69^***^ [1.28 to 2.22]	0.69 [0.46 to 1.01]	1.47^**^ [1.14 to 1.89]
Far-North	1.30 [0.96 to 1.75]	0.96 [0.65 to 1.41]	1.22 [0.93 to 1.61]
Littoral (without Douala)	1.39^*^ [1.05 to 1.83]	1.36^*^ [1.01 to 1.82]	1.39 [1.09 to 1.78]
North	0.60^*^ [0.40 to 0.89]	0.75 [0.50 to 1.12]	0.61^**^ [0.43 to 0.88]
North-West	1.05 [0.78 to 1.42]	0.71 [0.48 to 1.05]	1.00 [0.76 to 1.30]
West	1.72^***^ [1.30 to 2.28]	1.73^***^ [1.30 to 2.30]	1.74^***^ [1.35 to 2.22]
South	1.31 [0.99 to 1.73]	0.56^*^ [0.35 to 0.88]	1.10 [0.85 to 1.44]
South-West	1.48^*^ [1.08 to 2.02]	1.39 [0.94 to 2.04]	1.45^**^ [1.11 to 1.90]
Yaounde	1.39^*^ [1.06 to 1.83]	1.21 [0.89 to 1.66]	1.36^*^ [1.06 to 1.73]

APR, adjusted prevalence ratio.

Exponentiated coefficients; 95% CIs in brackets

^*^p≤0.05, ^**^p≤0.01, ^***^p≤0.001.

## Discussion

We estimated the prevalence and predictors of blood pressure and blood glucose screenings in Cameroon. We found that about two-thirds (59.9%) and a low proportion (29.6%) of Cameroonians had ever been screened for blood pressure and blood glucose, respectively. The results of low hypertension and diabetes screening uptake in the current study are consistent with studies elsewhere.^23,24^

Overall, gender was a key modifying factor. We found that age, education, marital status, household wealth index and region of residence were significant predictors for the gender-stratified modelling of both blood pressure and blood glucose screenings. Generally, females were screened for blood pressure and blood glucose more frequently than their male counterparts. This may be because females are believed to have a higher risk perception and display better health-seeking behaviour than males.^25^ Also, existing social systems, such as antenatal care services that incorporate routine blood pressure and blood glucose screenings, may contribute to the gender differences in screening uptake.^26^ Therefore, male-friendly interventions should incorporate blood pressure and glucose screenings to improve uptake among the general population. For both genders, blood glucose screening was significantly associated with blood pressure screening. Females who have been screened for blood pressure were more likely to have been screened for blood glucose.

Our study found a statistically significant association between age and blood pressure screening. Adults aged ≥25 y were more likely to undergo blood pressure screening compared with young adults aged <25 y. Blood pressure screening is implemented as part of routine health services or occupational services (or both), but also very often through opportunistic screening.^27^ Young adults tend to be healthier and are less likely to seek regular medical attention, decreasing their likelihood of hypertension and diabetes screenings. Conversely, older adults have the highest prevalence of hypertension and diabetes compared with young adults and therefore tend to be more aware about seeking regular medical attention.^28^

Place of residence was a predictor of blood glucose screening. Females resident in urban areas had a higher likelihood of undergoing blood glucose screening compared with their male counterparts. Urban residents were more likely to undertake blood pressure and blood glucose screenings compared with their rural counterparts. Urbanisation provides better access to healthcare services, education and social services.^14,29^ Also, better self-awareness among urban residents may lead to better access to preventive health services and disease management than among rural residents.^30^ Cameroon is a LMIC with about 44% of its people living in rural areas.^12^ Therefore, the government of Cameroon needs to invest in rural access to healthcare to achieve universal health coverage.^31^ Government support and the health system need to substantially fund these screening services.

Education was a significant predictor of blood glucose screening. People with at least secondary education were more likely to undergo blood glucose screening compared with their counterparts without formal education. This is possibly due to their knowledge of the benefits of early detection through screening. Having a higher than secondary education increased the likelihood of blood glucose screening among female subpopulations but not male subpopulations. Having at least a primary education increased the likelihood of blood pressure screening for both genders. Previous studies indicate that individuals lacking higher education are more likely to develop hypertension and diabetes.^32,33^ Educational level is regarded as an important health determinant, with potential gender-related differences.^34^ Health status in females is said to be more closely related to educational level, and the odds of experiencing diabetes or hypertension among women decreases with increasing educational level.^34^ Therefore, the government of Cameroon needs to prioritise education to achieve its multiple benefits, including screening uptake among Cameroonians.

Marital status was a predictor of blood pressure and blood glucose screenings, with females who were currently married being more likely to undergo both screenings. This may be partly because females who are currently married are more likely to be pregnant and thus utilise routine, mandatory antenatal screening services to prevent poor pregnancy outcomes.^35^ Also, higher rates of blood pressure and blood glucose screenings among currently married people could be explained by the mandatory health screening required by some religious groups before performing marriage ceremonies.^36^ A collaboration between public health officials and religious institutions could be harnessed to improve disease burden-reduction strategies. Evidence suggests that when one spouse has hypertension or diabetes, then the other appears to have a higher risk of developing the same disease.^37^ Therefore, couple-friendly health policy interventions could be developed to drive improved screening uptake by married couples. For example, antenatal care-friendly services may encourage men to accompany their partners to health facilities and be screened together for blood pressure and blood glucose.^38^

Household wealth predicted blood pressure and blood glucose screenings. We found that household wealth status was associated with an increased likelihood of hypertension and diabetes screenings. However, the magnitude of the effect was slightly higher for males than females with regard to blood glucose screening. Although men are generally at more risk of developing diabetes compared with their female counterparts,^39^ increasing household wealth status was associated with an increased likelihood of blood glucose screening for men in the current study. Financial assets are said to be associated with health due to the ability to afford those resources that protect and improve health,^40^ and males may be more conscious of their health if they are wealthy. For instance, lower socioeconomic status is related to higher blood pressure and blood glucose levels,^40^ and therefore public health officials need to devise pro-poor interventions to help improve screening uptake by poor households.

### Strengths and limitations 

One major strength of the current study was the use of a large, nationally representative survey dataset (2018 CDHS) based on a standardised methodology for analyses. Therefore, our findings can be generalised. Second, the study employed a complex sample analytical design to account for sampling units and weighting. Also, the study unmasked the high-risk population and region of the lowest proportions of blood pressure and blood glucose screenings in Cameroon. The main limitation of the current study is that we used secondary data that utilised a cross-sectional design. Hence, the associations observed in this study do not infer a causal relationship between the predictors and the outcome variables. Also, the study was restricted to variables available in the CDHS data.

### Conclusion

The current study identified vulnerable groups with low blood pressure and blood glucose screening uptake such as young adults, as well as those with little or no formal education. These people need to be considered when developing public health interventions and behavioural change strategies to improve their screening uptake to detect early, prevent or manage NCDs and their accompanying effects. Improving both wealth and educational status may be the critical pathway for the early detection and management of hypertension and diabetes. From the perspective of health policymaking, the results of this study call for effective strategies for early detection and management to curb hypertension, diabetes and their complications. The health system is severely underfunded with NCDs not being prioritised and there is a need for concerted efforts to help fight NCDs.

## Supplementary Material

ihab033_Supplemental_File

## Data Availability

The data used for this study can be freely obtained after a simple registration-access request to the DHS website at https://dhsprogram.com/data/dataset_admin/index.cfm.
