# Supplementary material for: Correlates of blood pressure and blood glucose screenings in Cameroon: insights from the 2018 Demographic and Health Survey
Source: Int Health. 2021 Jun 12;14(2):201–10. doi: 10.1093/inthealth/ihab033 (PMC8890807; doi:10.1093/inthealth/ihab033)
Supplement: ihab033_Supplemental_File [file ihab033_supplemental_file.docx]

**Supplementary Table: Weighted summary statistics of study variables (N = 20594)**

| **Correlates** | **Females** n= 13616 | **Males** n= 6978 | **Full sample** |
| --- | --- | --- | --- |
|  | n (%) | n (%) |  |
| **Blood pressure ever screened** |  |  |  |
| No | 4359 (32.0) | 3898 (55.9) | 8256 (40.1) |
| Yes | 9257 (68.0) | 3080 (44.1) | 12337 (59.9) |
| **Blood glucose ever tested** |  |  |  |
| No | 8919 (65.5) | 5576 (79.9) | 14495 (70.4) |
| Yes | 4697 (34.5) | 1402 (20.1) | 6099 (29.6) |
| **Age** |  |  |  |
| 15-24 | 5726 (42.1) | 2663 (38.2) | 8389 (40.7) |
| 25-34 | 4398 (32.3) | 1771 (25.4) | 6169 (30.0) |
| 35-44 | 2588 (19.0) | 1258 (18.0) | 3847 (18.7) |
| 45+ | 903 (6.6) | 1285 (18.4) | 2188 (10.6) |
| **Education** |  |  |  |
| None | 2778 (20.4) | 804 (11.5) | 3583 (17.4) |
| Primary | 3630 (26.7) | 1843 (26.4) | 5473 (26.6) |
| Secondary | 6158 (45.2) | 3575 (51.2) | 9733 (47.3) |
| Higher | 1049 (7.7) | 756 (10.8) | 1805 (8.8) |
| **Marital Status** |  |  |  |
| Never | 4692 (34.5) | 3360 (48.2) | 8052 (39.1) |
| Currently married | 7748 (56.9) | 3284 (47.1) | 11032 (53.6) |
| Previously married | 1175 (8.6) | 334 (4.8) | 1510 (7.3) |
| **Household wealth** |  |  |  |
| Poorest | 2239 (16.4) | 1076 (15.4) | 3314 (16.1) |
| Poorer | 2502 (18.4) | 1285 (18.4) | 3787 (18.4) |
| Middle | 2696 (19.8) | 1451 (20.8) | 4147 (20.1) |
| Richer | 2939 (21.6) | 1466 (21.0) | 4405 (21.4) |
| Richest | 3241 (23.8) | 1699 (24.3) | 4940 (24.0) |
| **Rural-Urban residence** |  |  |  |
| Urban | 7538 (55.4) | 3854 (55.2) | 11392 (55.3) |
| Rural | 6078 (44.6) | 3124 (44.8) | 9202 (44.7) |
| **Region of residence** |  |  |  |
| Adamawa | 630 (4.6) | 298 (4.3) | 928 (4.5) |
| Centre (without Yaounde) | 1350 (9.9) | 848 (12.1) | 2197 (10.7) |
| Douala | 1675 (12.3) | 896 (12.8) | 2572 (12.5) |
| East | 848 (6.2) | 402 (5.8) | 1250 (6.1) |
| Far-North | 2009 (14.8) | 976 (14.0) | 2985 (14.5) |
| Littoral (without Douala) | 507 (3.7) | 304 (4.4) | 811 (3.9) |
| North | 1720 (12.6) | 874 (12.5) | 2594 (12.6) |
| North-West | 882 (6.5) | 352 (5.1) | 1235 (6.0) |
| West | 1442 (10.6) | 714 (10.2) | 2156 (10.5) |
| South | 723 (5.3) | 387 (5.5) | 1111 (5.4) |
| South-West | 306 (2.2) | 140 (2.0) | 446 (2.2) |
| Yaounde | 1522 (11.2) | 787 (11.3) | 2309 (11.2) |
